# Neurobiological Correlation Between Autism Spectrum Disorder and Anorexia Nervosa in Children

**DOI:** 10.1192/j.eurpsy.2024.935

**Published:** 2024-08-27

**Authors:** K. Shah, P. Reddy, A. Giri, S. Srinivas

**Affiliations:** ^1^ Wake Forest University; ^2^ Baptist Health - UAMS Medical Education Program PGY-4 Psychiatry Resident; ^3^Dhaka University, Winston-Salem; ^4^Baptist Health and the University of Arkansas for Medical Sciences (BH-UAMS), Little Rock, United States

## Abstract

**Introduction:**

Anorexia Nervosa (AN) is common in adolescents and has a high mortality and morbidity rate with a lifetime prevalence of 0.5% to 2%.1,2 We aim to review the neurobiology correlation of Anorexia Nervosa in Autism Spectrum Disorder as they are often associated together.

**Objectives:**

1. Understand the correlation between the neurobiology of Autism Spectrum Disorder (ASD) and Anorexia Nervosa.

2. Assess the association and prevalence of Anorexia nervosa in the ASD population.

3. To focus on the implications for the pathogenesis of Anorexia Nervosa and treatment of this disorder in the ASD population.

**Methods:**

We searched PubMed, APA PscyINFO, Embase, CINAHL, and Google scholar databases with the keywords Autism Spectrum Disorder AND Anorexia Nervosa and included 6 relevant human studies out of 187 published in English.

**Results:**

Neilson et al. studied the outcome of ASD in teenage onset AN, and a statistically significant negative dose-response relationship is found in all the 3 Morgan-Russell Outcome Assessment Schedule (MROAS) domains in stable ASD over time, and the results on the subscales ‘mental state,’ ‘psychosexual state’ and ‘socio-economic state, “personal contacts,’ ‘social activities’ and ‘employment record.’3 The outcome of AN onset in adolescence is generally favorable regarding mortality and the persistence of eating disorders in adulthood. A study by Pruccoli et al. noted a high prevalence of ASD traits in a group of young AN patients, predominantly seen in 4 specific EDI-3 subscales and independent of BMI.4 Margari et al. found only AN diagnosis had a statistically significant difference (p = 0.04) in females vs. males when comparing sex differences for comorbidities.5

**Conclusions:**

Morphological changes in brain areas are linked to social cognition and increase the risk of eating disorders in ASD. We recommend future studies with robust study design to explore the full spectrum of pathogenesis and association of AN in ASD.

**Disclosure of Interest:**

None Declared

